# Summer paleohydrology during the Late Glacial and Early Holocene based on δ^2^H and δ^18^O from Bichlersee, Bavaria

**DOI:** 10.1038/s41598-023-45754-4

**Published:** 2023-10-28

**Authors:** Maximilian Prochnow, Paul Strobel, Marcel Bliedtner, Julian Struck, Lucas Bittner, Sönke Szidat, Gary Salazar, Heike Schneider, Sudip Acharya, Michael Zech, Roland Zech

**Affiliations:** 1 Chair of Physical Geography, Institute of Geography, Friedrich-Schiller-Universität Jena, Jena, Germany; 2 Heisenberg Chair of Physical Geography with Focus on Paleoenvironmental Research, Institute of Geography, Technische Universität Dresden, Dresden, Germany; 3 Department of Chemistry, Biochemistry and Pharmaceutical Sciences and Oeschger Centre for Climate Change Research, University of Bern, Bern, Switzerland

**Keywords:** Palaeoclimate, Biogeochemistry

## Abstract

Isotope-based records provide valuable information on past climate changes. However, it is not always trivial to disentangle past changes in the isotopic composition of precipitation from possible changes in evaporative enrichment, and seasonality may need to be considered. Here, we analyzed δ^2^H on *n*-alkanes and δ^18^O on hemicellulose sugars in sediments from Bichlersee, Bavaria, covering the Late Glacial and Early Holocene. Our δ^2^H_*n*-C31_ record documents past changes in the isotopic composition of summer precipitation and roughly shows the isotope pattern known from Greenland. Both records show lower values during the Younger Dryas, but at Bichlersee the signal is less pronounced, corroborating earlier suggestions that the Younger Dryas was mainly a winter phenomenon and less extreme during summer. δ^18^O_fucose_ records the isotopic composition of the lake water during summer and is sensitive to evaporative enrichment. Coupling δ^2^H_*n*-C31_ and δ^18^O_fucose_ allows calculating lake water deuterium-excess and thus disentangling changes in the isotopic composition of precipitation and evaporative enrichment. Our deuterium-excess record reveals that the warm Bølling–Allerød and Early Holocene were characterized by more evaporative enrichment compared to the colder Younger Dryas. Site-specific hydrological conditions, seasonality, and coupling δ^2^H and δ^18^O are thus important when interpreting isotope records.

## Introduction

High-altitude ecosystems, such as the European Alps, are highly sensitive to climate change^1–4^. Hydrological aspects like melting glaciers and changing precipitation pattern will increase the risk of landscape destabilization, water scarcity and more frequent flooding in the future^5–7^. In this context, paleoclimate studies can provide valuable information to better understand past and predict future climate-landscape interactions^8^. In particular the Late Glacial–Early Holocene transition is of interest because it is known for its rapid climate changes and has been intensively investigated^9,10^. The Late Glacial comprises the warm Bølling–Allerød interstadial (starting ~ 14.7 ka BP) and the cold Younger Dryas stadial (from ~ 12.8 to 11.7 ka BP)^11,12^. Paleoclimate information from the Alps during that time is inferred inter alia from stable oxygen isotope records (δ^18^O) derived from lake sediments^13–17^ and speleothems^18,19^. Those δ^18^O records mostly resemble the δ^18^O records from Greenland ice cores^20^ and are often interpreted to document past temperature changes. However, the interpretation of δ^18^O alone can be challenging because various factors can influence the isotopic signal, which are difficult to disentangle. Those include changes in atmospheric circulation, precipitation, seasonality, carbonate chemistry, and regarding lake sediments, evaporative enrichment of lake water^18,21–23^. Past changes in evaporative enrichment could have played an important role due to rapidly changing warm and cold conditions during the Late Glacial–Early Holocene transition. Although this effect can complicate the interpretation of δ^18^O records, evaporative enrichment itself provides very valuable hydrological information, e.g., about wet or dry conditions. This is particularly interesting in view of the scarce and controversial data concerning the hydroclimatic development during the Late Glacial. Pollen from terrestrial and marine sediments, for example, are often interpreted to indicate warm and humid conditions during the Bølling–Allerød and Early Holocene^24,25^, but drier conditions have also been suggested^26,27^. For the Younger Dryas, there is also no consensus, although first attempts of paleohydrological reconstructions have been made using novel biomarker and isotope analyses^28–30^.

Over the last few years, compound-specific hydrogen (δ^2^H) and oxygen (δ^18^O) isotope analyses on biomarkers, i.e., molecular fossils, have been developed. Long-chain *n*-alkanes (*n*-C_29_ and *n*-C_31_), for example, are leaf waxes produced by higher terrestrial plants. Their δ^2^H signal mainly reflects the isotopic composition of the local precipitation but can be modulated by transpirative enrichment of the leaf water and biosynthetic fractionation^31–34^. Shorter-chain *n*-alkanes (e.g., *n-*C_21_ and *n-*C_23_), on the other hand, are produced by aquatic organisms and incorporate the δ^2^H signal of the lake water, i.e., depending on the hydrological setting, evaporation may lead to isotopic enrichment^35–39^. Like δ^2^H from *n*-alkanes, δ^18^O from hemicellulose sugars provides valuable paleohydrological information^34,35,40,41^. Hemicellulose sugars are also produced by terrestrial (i.e., arabinose) and aquatic (i.e., fucose) sources^42^. Their δ^18^O signal mainly reflects the isotopic composition of the local precipitation (arabinose) and the lake water (fucose) modulated by evapo(transpi)rative enrichment and biosynthetic fractionation^32,33,41,43^. The “coupled isotope approach”, also dubbed “paleohygrometer approach”, combines δ^2^H_*n*-alkane_ and δ^18^O_sugar_ analyses to reconstruct deuterium excess (*d*-excess), which can be used as a proxy for evapo(transpi)rative enrichment^30,35,43,44^. This approach has been tested and applied successfully in several studies to quantify the evapo(transpi)rative enrichment of leaf water^30,35,40,43–45^ and lake water^35^.

So far, very few studies have applied biomarker and compound-specific isotope analyses in the European Alps during the Late Glacial-Early Holocene transition^23,46^, but they have great potential to investigate the common notion of warm and humid interstadials, versus cool and dry stadials. Therefore, the aim of this study is to establish a high-resolution δ^2^H_*n*-alkane_ record for the Late Glacial–Early Holocene sediments from Bichlersee (Bavaria, Germany) and complement this with δ^18^O_sugar_ analyses. Specifically, we aim to (1) identify the sources of the biomarker compounds (terrestrial versus aquatic) and (2) discuss the role of evaporative and transpirative enrichment of the lake and leaf water, respectively. Afterwards, (3) the coupled isotope approach will be applied to calculate the deuterium excess as a proxy for lake water evaporation.

## Geographic setting of Bichlersee

**Figure 1:**
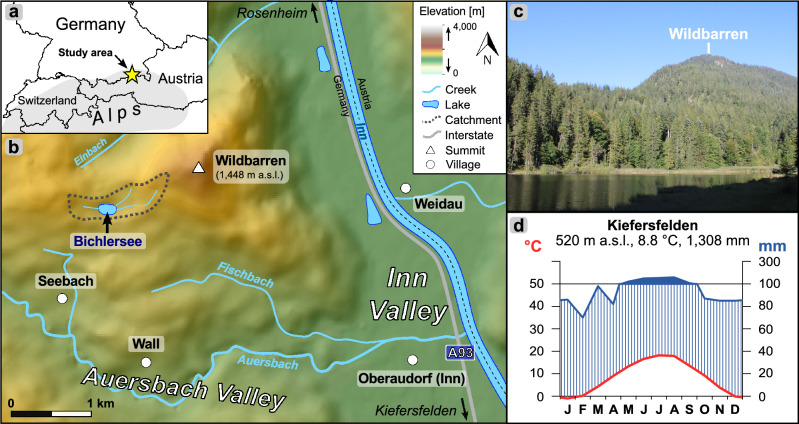
Geographic setting of Bichlersee. (**a**) Overview map showing the location of the study site. (**b**) Topographical map of the Bichlersee area (Data: Copernicus EuropeDEM 1.1). (**c**) Photograph of Bichlersee and the forested Wildbarren (Photo: M. Prochnow). (**d**) Climate diagram from Kiefersfelden illustrating local climate conditions (1991–2020)^48^. The maps in (**a**) and (**b**) were created with ArcGIS Pro 2.9.2 (www.esri.com/de-de/arcgis/products/arcgis-pro) and all image labels were added using Inkscape 1.2.2 (www.inkscape.org). The climate diagram in (**d**) was created with Climatol 4.0.5 for R (www.climatol.eu).

Bichlersee (See = lake) is located in the Northern Limestone Alps ~ 5 km northwest of Oberaudorf in the lower Inn Valley (Fig. 1a,b). It is situated in a small karst depression at 960 m a.s.l. west of the Wildbarren (1,448 m a.s.l.). The circular lake has an area of 0.01 km^2^ and a maximum water depth of 11 m^47^. The catchment comprises an area of ~ 0.5 km^2^ including swampy areas along the lake shore and three small creeks (Fig. 1b,c). The surrounding slopes are covered by dense mountain forest consisting of *Picea*, *Abies*, and *Fagus*. The study site is affected by the Westerly circulation system bringing moisture from the Atlantic Ocean to the Alps. Mean annual temperature (MAT) in Kiefersfelden (~ 7.5 km to the south, 520 m a.s.l.) is 8.8 °C and mean annual precipitation (MAP) is 1308 mm (Reference period 1991–2020, Fig. 1d)^48^. The isotopic composition of precipitation (δ^2^H_p_, δ^18^O_p_) shows a strong seasonal variability: Summer precipitation is isotopically enriched (− 34‰ for δ^2^H_p_ and − 5.2‰ for δ^18^O_p_ in July), whereas winter precipitation is generally strongly depleted with − 108‰ for δ^2^H_p_ and − 14.8‰ for δ^18^O_p_ in January^49,50^. The isotopic composition of precipitation is very heterogenous across the European Alps and influenced by various effects, including different moisture sources. However, the most important effect explaining ~ 70% of the seasonal variability in δ^18^O_p_ and δ^2^H_p_ is temperature^23,51^.

## Results and discussion

### Lithology and chronology

**Figure 2:**
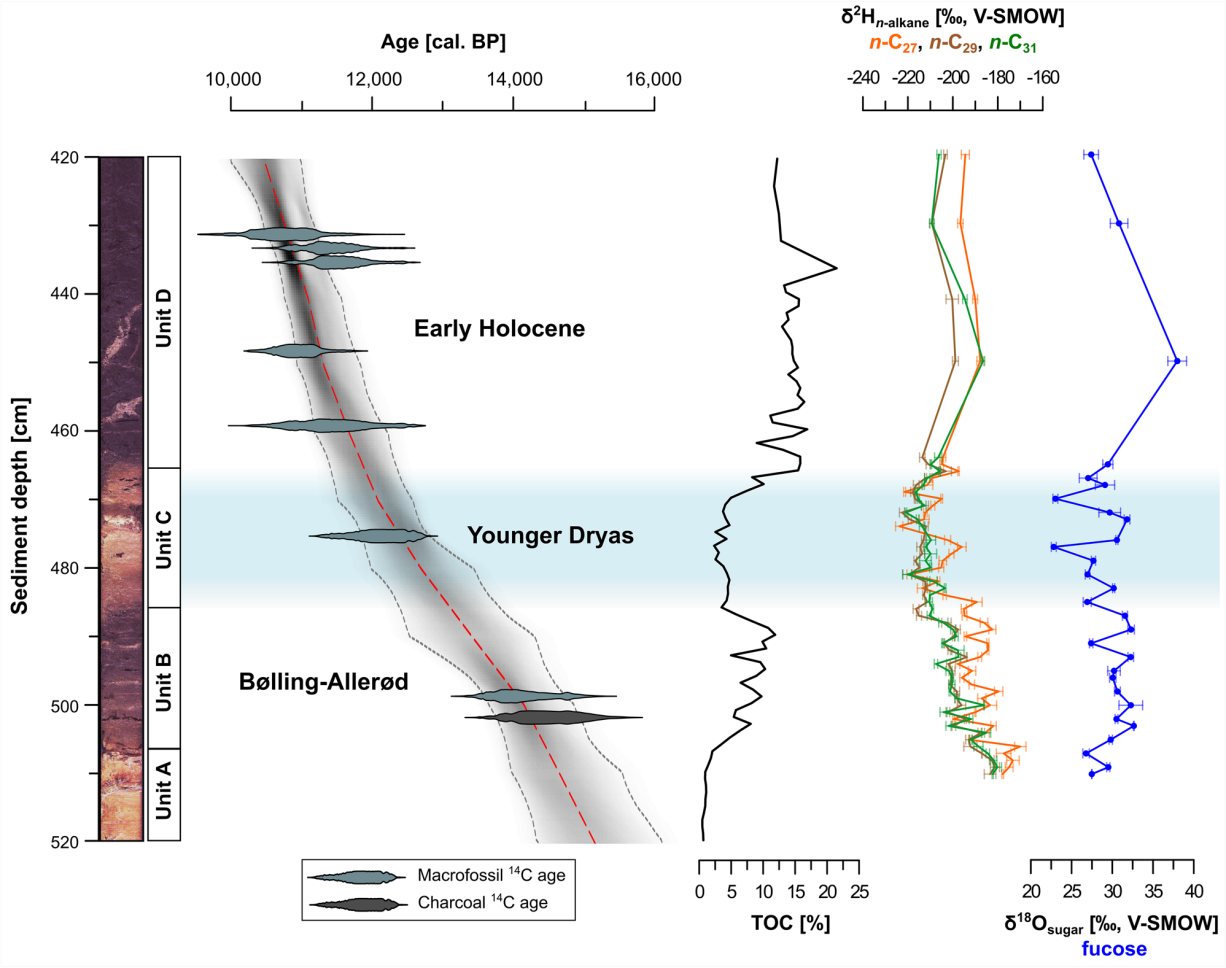
Core photograph, age depth model, and results of geochemical as well as stable isotope analyses for the Late Glacial–Early Holocene part of our core from Bichlersee. The graphic was created with Inkscape 1.2.2 (www.inkscape.org).

In 2014, we recovered a 9.4 m long sediment core from Bichlersee. For this study, we focus on the sediments between 520 and 420 cm, which comprise the Late Glacial and transition into the Early Holocene. Unit A from 520 to 508 cm shows light colored sediments with TOC contents below 5% (Fig. 2). Unit B (508–487 cm) consists of dark sediments with high TOC contents up to 13%. Unit C (487–467 cm) is then again characterized by lighter colors and lower TOC contents, while Unit D (above 467 cm) is dark-brown with high TOC contents (15%). The two bright mottles in Unit D (458 cm, 445 cm) probably indicate disturbance of the sediment and were avoided during sampling. The rest of the core is finely layered.

Radiocarbon ages from eight organic macrofossils and charcoal particles (BS_1 to BS_8) provide a very consistent and robust chronological control (Fig. 2, Table 1). Bayesian age-depth modeling gives a basal median age of 15,118 ^+980^/_−860_ cal. BP. The age-depth model is corroborated by the pattern of TOC, suggesting that Unit A reflects the Oldest Dryas, Unit B the Bølling–Allerød, Unit C the Younger Dryas and Unit D the Early Holocene^12,20^. TOC reflects changes in productivity during these periods, with lower TOC during the Younger Dryas because of cooler temperatures and reduced bioproductivity. This seems justified given that similar TOC pattern during the Late Glacial have been reported from other lakes, for example the pre-alpine lakes Steißlingen^52^ and Mondsee^15^. A tree cone ^14^C age (12,130 ^+600^/_−770_ cal. BP) falls directly into the Younger Dryas, and the Early Holocene is covered by several consistent terrestrial macrofossil ^14^C ages ranging from 11,430 ^+1160^/_−930_ to 10,780 ^+1040^/_−880_ cal. BP (Fig. 2).

**Table 1:** ^14^C dating results for Bichlersee.

Labcode	ID	Sample type	Depth (cm)	Carbon mass (µg)	^14^C age (a BP)	Calibrated age (cal. BP, 2σ range)	Calibrated median age (cal. BP, 2σ range)
BE-4401.1.1	BS_1	Leaf	431	48.0	9458 ± 314	9896–11,814	10,780 ^+1030^/_−880_
BE-4407.1.1	BS_2	Leaf	433	59.3	9869 ± 219	10,593–12,427	11,380 ^+1050^/_−720_
BE-4406.1.1	BS_3	Bark	435	58.9	9956 ± 221	10,773–12,460	11,520 ^+940^/_−820_
BE-4414.1.1	BS_4	Bark	447	54.8	9618 ± 215	10,299–11,687	10,950 ^+740^/_−650_
BE-4403.1.1	BS_5	Leaf	458	49.7	9882 ± 326	10,507–12,595	11,430 ^+1160^/_−920_
BE-4400.1.1	BS_6	Cone	474	44.5	10,341 ± 226	11,358–12,724	12,130 ^+590^/_−770_
BE-4404.1.1	BS_7	Leaf	499	31.5	12,083 ± 274	13,455–15,062	14,100 ^+960^/_−640_
BE-4412.1.1	BS_8	Charcoal	501	47.0	12,336 ± 288	13,608–15,395	14,510 ^+880^/_−900_

### ***n***-Alkanes and compound-specific δ^2^H

*n*-Alkanes were obtained in sufficient amounts for high-resolution compound-specific δ^2^H analyses only for the Late Glacial and Early Holocene part of the core, and we had to focus on the most abundant homologues *n*-C_27_ to *n*-C_31_. δ^2^H ranges from − 223.7 to − 170.1‰ for *n*-C_27_, from − 222.5 to − 181.3‰ for *n*-C_29_, and from − 220.7 to − 180.3‰ for *n*-C_31_, respectively (Fig. 2). *n*-Alkanes are generally more enriched in Unit B and Unit D, and more depleted in Unit C. δ^2^H_*n*-C29_ and δ^2^H_*n*-C31_ show a very similar pattern, whereas δ^2^H_*n*-C27_ is more variable and more positive, which indicates different sources and different paleohydrological implications.

In general, *n*-C_31_ and *n*-C_33_ tend to be more abundant in grasses and herbs, whereas *n*-C_27_ and *n*-C_29_ are preferentially produced by deciduous trees and shrubs^53^. With the beginning of the Bølling–Allerød, the vegetation was dominated by *Betula* and *Pinus* forests persisting at least into the Preboreal^54,55^. Such forests have a dense grassy understory vegetation, as we can confirm by high abundances of Poaceae in three exemplarily investigated pollen samples within the Bølling–Allerød (7–11%; see Supplementary Fig. S3). The most abundant pollen are *Betula pendula* with 25–43% and *Pinus sylvestris* with 43–60%. We can therefore assume that *n*-C_31_ is primarily derived from grasses, because coniferous trees produce no long-chain *n*-alkanes, yet *Pinus* growing in the montane zone of the Alps is known to synthesize only very low amounts of *n*-alkanes^46^. While a distinct exclusive attribution is not possible, we can use the δ^2^H_*n*-C31_ signal as the best available record for terrestrial grasses, which is very relevant with regard to the fact that leaf waxes from grasses are less affected by transpirative enrichment than trees and shrubs^56^. This is due to the so-called ‘damping-effect’^45,56^, i.e., grasses grow through the less exposed intercalary meristems^57^ and are thus less affected by transpirative leaf water enrichment^58^. δ^2^H_*n*-C31_ is therefore assumed to mainly reflect changes in the isotopic composition of precipitation during the growing season.

Our δ^2^H_*n*-C31_ record is in good agreement with results from Meerfelder Maar^29^ and Gemündener Maar^30^ in northern Germany, providing a coherent pattern between the existing terrestrial δ^2^H_*n*-alkane_ records (Fig. 4e). This pattern mostly follows δ^18^O and δ^2^H in Greenland ice cores^20^ (Fig. 4f,g), where the reconstructed isotopic composition of precipitation is traditionally explained to reflect northern hemispheric temperature changes^11^ (i.e., the typical stadial-interstadial pattern): more enriched values during the Bølling–Allerød interstadial and Early Holocene are related to warmer temperatures, interrupted by more depleted values during the cooler Younger Dryas stadial (Fig. 4a,f). Detailed comparison reveals that δ^2^H in Greenland (Fig. 4g) varies by ~ 40‰ during the Bølling–Allerød and drops markedly into the Younger Dryas^59^, while δ^2^H_*n*-C31_ at Bichlersee decreases rather gently by only ~ 20‰ (Fig. 4a). While temperature and related isotope changes were probably more pronounced in Greenland than at lower latitudes like the Alps^60^, seasonality might have a greater influence on the isotopic signal at our site^23^. Because temperatures dropped much less in summers than winters during the Younger Dryas^61,62^, our (summer) δ^2^H_*n*-C31_ record does not show the pronounced (annual) stadial-interstadial signal as the ice cores from Greenland.

We suggest that the more variable and more positive δ^2^H_*n*-C27_ signal (up to ~ 20‰) can be explained by the fact that *n*-C_27_ is to a higher degree derived from deciduous trees and shrubs than *n*-C_29_ or even *n*-C_31_. *n*-C_27_ is therefore more affected by leaf water transpirative enrichment (Fig. 4a). *Betula pendula* produces high amounts of *n*-C_27_^46,53,63^ and its occurrence at Bichlersee is documented by high pollen abundances since the Bølling–Allerød (see Supplementary Fig. S3). Vegetation changes such as an expansion of grasses during the Younger Dryas^64^ might affect the isotopic composition of *n*-alkanes, i.e. leading to more depleted δ^2^H, but light *Betula* and *Pinus* forests persisted throughout the Younger Dryas at sites < 1700 m a.s.l.^26,65^. This is corroborated by relatively constant relative abundances of *n*-C_27_ and *n*-C_31_ during the Bølling–Allerød and Younger Dryas at Bichlersee (see Supplementary Fig. S4).

Recent studies provide evidence that long-chain *n*-alkanes including *n*-C_27_ can also originate from aquatic plants^66–68^. In fact, the relative concentration of *n*-C_27_ positively correlates with *n*-C_25_ (*r* = 0.8), but negatively with *n*-C_29_ and *n*-C_31_ (*r* =  − 0.6 and − 0.4) in our dataset (Table 2). We therefore assume that *n*-C_27_ has a mixed origin from aquatic and terrestrial sources and therefore partly reflects lake water, not just leaf water δ^2^H.

**Table 2:** Pearson *r* correlations between relative abundances of *n*-alkanes.

	***n*-C**_**21**_	***n*-C**_**23**_	***n*-C**_**25**_	***n*-C**_**27**_	***n*-C**_**29**_	***n*-C**_**31**_	***n*-C**_**33**_
*n*-C_21_	1.0						
*n*-C_23_	**0.70**	1.0					
*n*-C_25_	0.0	**0.30**	1.0				
*n*-C_27_	− 0.2	0.1	**0.8**	1.0			
*n*-C_29_	− 0.2	− **0.50**	− **0.45**	− **0.59**	1.0		
*n*-C_31_	− **0.55**	− **0.84**	− **0.67**	− **0.45**	**0.48**	1.0	
*n*-C_33_	− **0.44**	− **0.56**	− **0.43**	− 0.1	− 0.2	**0.73**	1.0

Bold values are significant (*p* < 0.05) within the 95% confidence interval.

### Hemicellulose sugars and compound-specific δ^18^O

**Figure 3:**
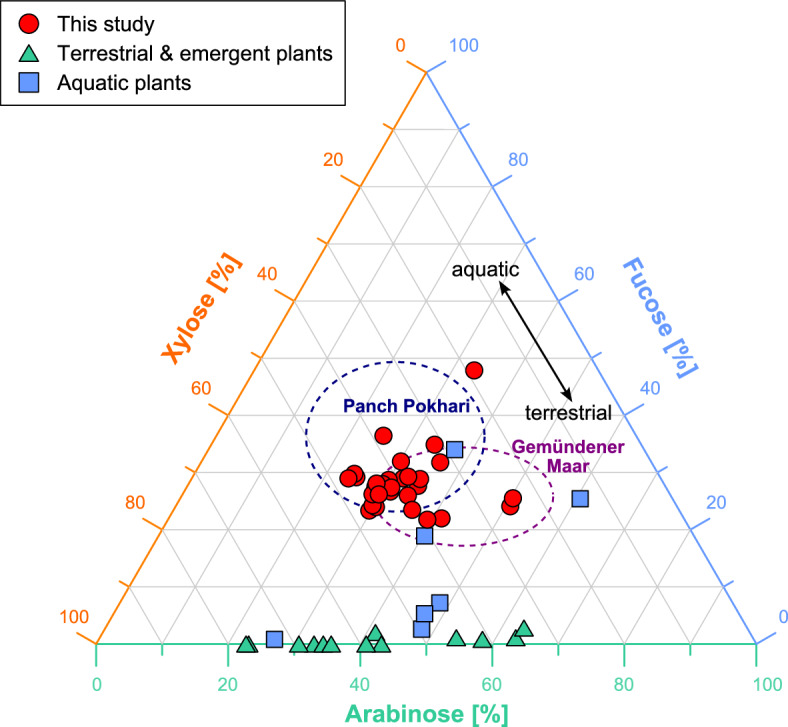
Ternary diagram of relative abundances of hemicellulose sugars from Bichlersee. The diagram shows relative abundances of arabinose, fucose, and xylose in the Bichlersee samples (red dots) and data from previous studies: emergent, terrestrial and submerged plants from Bichlersee^42^, Panch Pokhari^69^ and Gemündener Maar^30^. The graphic was created with Inkscape 1.2.2 (www.inkscape.org).

A ternary diagram (Fig. 3) with the relative concentrations of arabinose, fucose and xylose shows that our samples from Bichlersee have a relative abundance of ~ 30% fucose, which is comparable to lake sediment samples from Panch Pokhari, Nepal^69^, Gemündener Maar, Germany^30^, as well as some submerged plants from Bichlersee^42^. Samples from emergent and terrestrial plants from Bichlersee, on the other hand, show almost no fucose^42^. Fucose is known to be highly abundant in zooplankton, phytoplankton and aquatic bacteria^70^, but low in terrestrial plants^42,69^. It is thus a predominantly aquatic compound. Arabinose and xylose, on the other hand, are of mixed aquatic and terrestrial origin at Bichlersee. In the following, we will therefore focus on δ^18^O_fucose_ as a proxy for lake water δ^18^O, as recently suggested by Bittner, et al.^41^ who showed that δ^18^O_fucose_ agrees very well with δ^18^O_diatom_.

**Figure 4:**
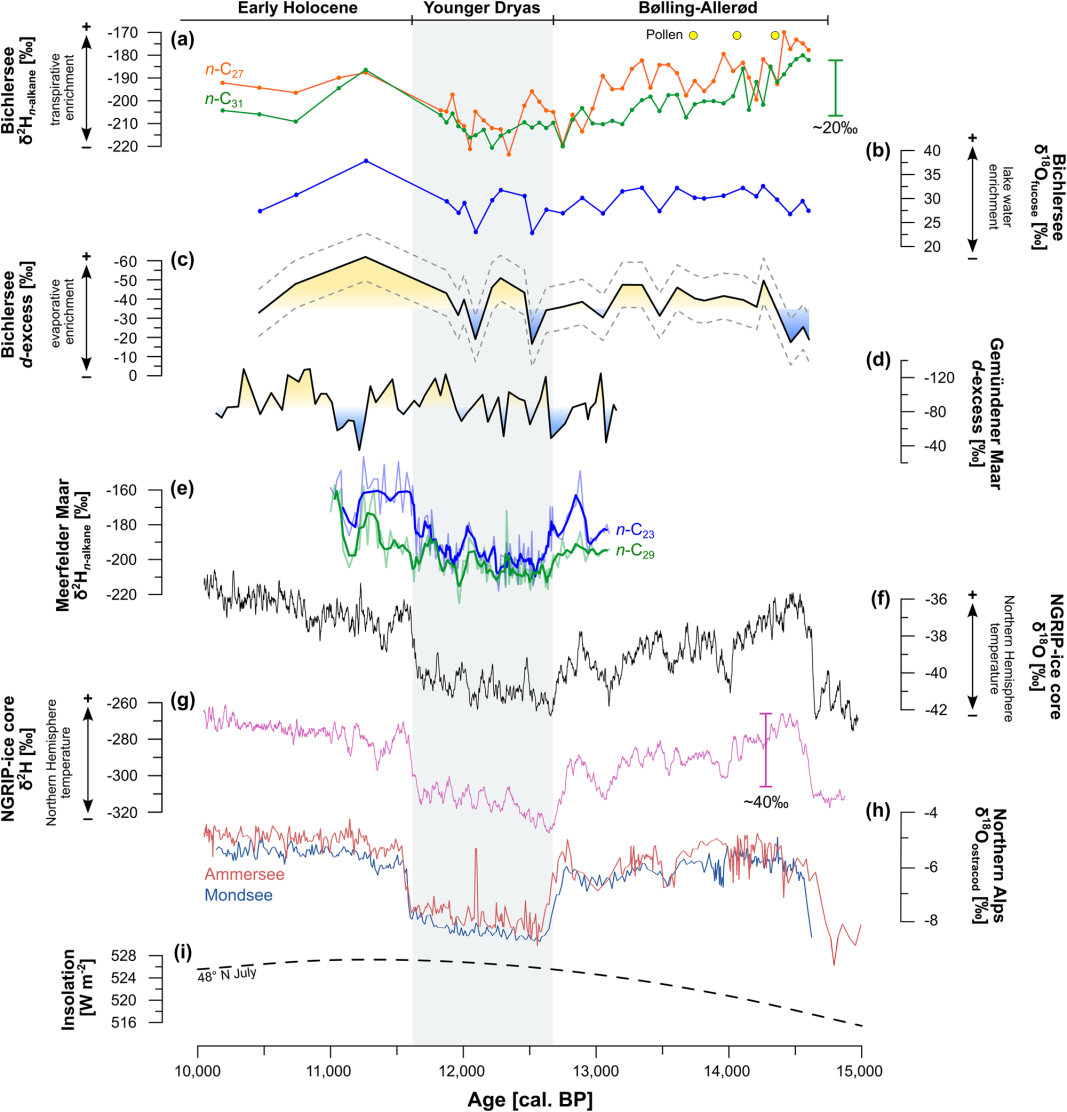
Late Glacial–Early Holocene compilation of (**a**) δ^2^H_*n*-alkane_ and (**b**) δ^18^O_fucose_ from Bichlersee. The estimated *d*-excess for Bichlersee is plotted in (**c**) and the *d*-excess from Gemündener Maar^30^ is shown in (**d**). δ^2^H_*n*-alkane_ from Meerfelder Maar^29^ is given in (**e**), (**f**) and (**g**) illustrate ice core δ^18^O and δ^2^H from NGRIP (Greenland)^20,59^ reflecting northern hemispheric temperature, (**h**) reflects ostracod δ^18^O from Ammersee^14^ and Mondsee^15^, and (**i**) shows the July summer insolation at 48° N^78^. The graphic was created with Inkscape 1.2.2 (www.inkscape.org).

δ^18^O_fucose_ ranges from 22.8 to 37.8‰ and is highly variable (Fig. 2). It shows the stadial-interstadial pattern, i.e., more positive values during the Bølling–Allerød and Early Holocene interrupted by more negative values during the Younger Dryas (Fig. 4b). However, the pattern of our δ^18^O_fucose_ record reveals a high variability and is less distinct compared to our δ^2^H record, Greenland δ^18^O (Fig. 4f), and δ^18^O from other alpine and pre-alpine lakes, for example, Lake Ammersee^14^ and Mondsee^15^ (Fig. 4h). These differences are partly related to specific hydrological settings like surface area, water volume and lakebed geometry. Ammersee, for instance, located 80 km northwest of Bichlersee near Munich, has a large catchment area and a large and deep water body. δ^18^O on benthic ostracods from Ammersee thus likely represents an annual lake water signal^17^ and is less sensitive to the effect of evaporative enrichment^14^. Bichlersee and its catchment, on the other hand, are very small and particularly sensitive to record the isotopic signal of summer precipitation. As mentioned above for δ^2^H, the Younger Dryas was mainly a winter temperature phenomenon^61,62^, so one can hypothesize that—analogously to our δ^2^H_*n*-C31_ record—a summer isotope record like δ^18^O_fucose_ shows a less pronounced stadial-interstadial pattern than Greenland. Moreover, the high variability of our δ^18^O_fucose_ record can at least partly be explained with the high sensitivity of Bichlersee for evaporative enrichment. Bichlersee has a closed basin (although groundwater outflow is likely), so the ratio of precipitation to evaporation (P/E) in summer strongly controls δ^18^O of the lake water^41^ and the fucose. Several studies have shown that lake water ^18^O enrichment varies highly between lakes and can reach up to several per mille (~ 5‰ in δ^18^O) even under todays humid climate conditions in Germany and the Alps^16,21,71,72^. Evaporative enrichment can best be discussed not just looking at one isotope record, but combining δ^2^H and δ^18^O.

### Calculating deuterium excess by coupling of δ^18^O_sugar_ and δ^2^H_***n-***alkane_

**Figure 5:**
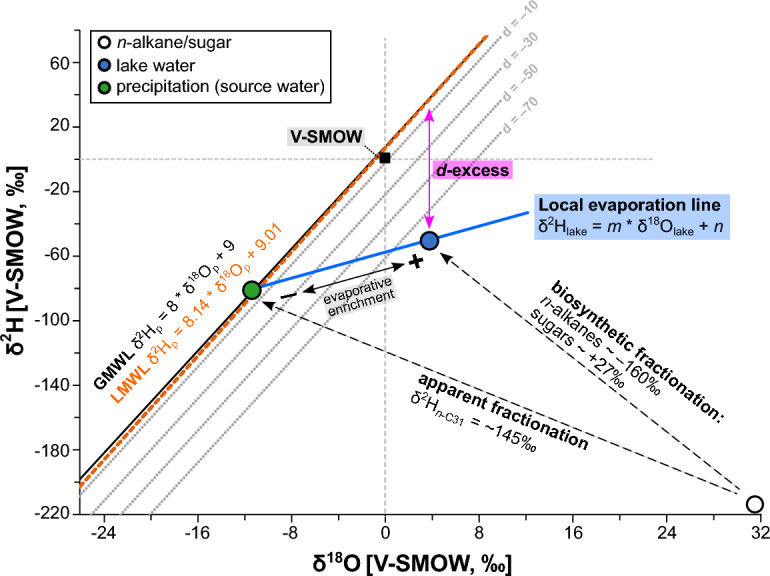
Conceptual framework of the coupled isotope approach after Hepp et al.^35^. The graphic was created with Inkscape 1.2.2 (www.inkscape.org).

Based on the discussion above, δ^2^H_*n*-C31_ and δ^18^O_fucose_ are the best available terrestrial and aquatic isotope proxies, respectively. Based on these, one can calculate lake water deuterium excess (*d*-excess), an important proxy for evaporative enrichment, using an adapted version of the “coupled isotope approach” described by Hepp et al.^35^. As illustrated in the δ^18^O–δ^2^H-diagram (Fig. 5), the basic assumption of this approach is that the isotopic composition of lake water can be calculated by applying the specific biosynthetic fractionation for *n*-alkanes and sugars. Lake water then plots on a local evaporation line (LEL). When the degree of evaporative enrichment is low, the isotopic composition of the lake water plots close to the starting point of the LEL on the Global Meteoric Waterline (GMWL, Eq. 1)^60^:

1 $$\updelta ^{2} {\text{H}} = 8*\updelta ^{18} {\text{O}} + 10$$

With increasing degree of evaporative enrichment, the distance of lake water to the GMWL increases following the LEL. Thus, deuterium excess (*d*-excess) of the lake water is defined as distance from the GMWL, with more negative values indicating stronger evaporative enrichment. Instead of the GMWL, site-specific Local Meteoric Waterlines (LMWL, Eq. 2) can be used, in our case

2 $$\updelta ^{2} {\text{H}} = 8.14*\updelta ^{18} {\text{O}} + 9.05$$

based on data from the climate station Garmisch-Partenkirchen^73^.

Applying the coupled isotope approach, we first subtracted the apparent fractionation − 145 ± 12‰ for Central Europe^34^ from our δ^2^H_*n*-C31_ values in order to estimate δ^2^H of precipitation (δ^2^H_p_) used for biosynthesis (Fig. 5). As described in “*n*-Alkanes and compound-specific δ^2^H” section, this is based on the assumption that δ^2^H_*n*-C31_ is primarily derived from grasses, which are less affected by transpirative enrichment and therefore changes in δ^2^H_*n*-C31_ mainly record changes in the isotopic composition of precipitation. δ^2^H_p_ is then used to calculate δ^18^O_p_ based on the LMWL (Eq. 2). For δ^18^O_sugar_, the biosynthetic fractionation factor is ~ 27‰^74,75^, which can be used to calculate lake water δ^18^O (δ^18^O_lake_). The corresponding δ^2^H_lake_ values are derived with local evaporation lines described by Eq. (3):

3 $$\updelta ^{{2}} {\text{H}}_{{{\text{lake}}}} = m*\updelta ^{{{18}}} {\text{O}}_{{{\text{lake}}}} + n$$

where *m* is the temperature-dependent slope and *n* the intercept with δ^2^H (Fig. 5). A more detailed methodological description of the *d*-excess estimation and its potential limitations is provided in the Supplementary section S1.

In general, our *d*-excess record has a very similar pattern compared to δ^18^O_fucose_ (note the reversed axis in Fig. 4c, r =  − 0.87, *p* = 6.86 × 10^−10^). This illustrates the high sensitivity of δ^18^O to evapo(transpi)rative enrichment compared to δ^2^H, which has previously been concluded from transect and growth chamber studies^34,56,76,77^. Analyzing δ^18^O_sugar_ and calculating *d*-excess thus has a major added value compared to only analyzing δ^2^H on *n*-alkanes, as it allows to disentangle past changes in the isotopic composition of precipitation and evaporative enrichment.

Our *d*-excess record shows a long-term trend towards more lake water enrichment from the Late Glacial to the Early Holocene, which can possibly be explained with increasing summer insolation^78^ (Fig. 4i) and related evaporation. More importantly, the Bølling–Allerød and Early Holocene are characterized by enhanced enrichment compared to the Younger Dryas. This makes sense in view of the higher temperatures driving more evaporation. Our results agree well with a pollen study by Ammann, et al.^26^ who reconstructed higher evapo(transpi)ration during the Bølling–Allerød at Gerzensee in Switzerland. Similarly, Litt et al.^27^ suggested more arid conditions during the Early Holocene based on pollen from lake Holzmaar in Germany. We can further compare our record with the *d*-excess from the Gemündener Maar^30^, the only other available Late Glacial *d*-excess record for Central Europe (Fig. 4d). There, *d*-excess was derived by a coupled isotope approach based on leaf water δ^18^O and δ^2^H. Both records show high variability, but no evidence for an overall dry Younger Dryas^28,29^. Aridity north of the Alps during the Younger Dryas was explained by a southward migration of the Westerlies in relation to cooling and enhanced sea-ice cover in the North Atlantic^79^. This shift occurred most likely during winter and lead to increased winds and hence dryness in Central Europe, but as it can be expected from variable sea-ice cover in the North Atlantic^80^, this shift was apparently not persistent throughout the Younger Dryas^81,82^. It can be moreover only speculated how a shift of the Westerlies affected summer hydrology at Bichlersee, and while our study emphasizes the relevance of seasonality to this issue, we believe that more paleohydrology records are strongly needed to further confirm these atmospheric mechanisms.

## Conclusion

This study presents the first lacustrine δ^2^H_*n*-alkane_ record from the Northern European Alps covering the Late Glacial–Early Holocene transition (~ 15–10 ka BP). We complemented this with δ^18^O_sugar_ analyses to explore the potential of combining δ^2^H and δ^18^O for paleohydrological reconstructions at Bichlersee. In view of the scarce and controversial data concerning the hydroclimatic development during the Late Glacial, our study aimed to test the general notion of warm and humid interstadials, versus cool and dry stadials.

δ^2^H_*n*-C31_ primarily reflects changes in the isotopic composition of summer precipitation because *n*-C_31_ is predominantly produced by grasses which are not particularly sensitive to leaf water transpirative enrichment. Our δ^2^H_*n*-C31_ record shows more negative values during the Younger Dryas compared to the Bølling–Allerød and Early Holocene. It thus agrees with other published leaf-wax records and isotope records from Greenland ice cores showing the typical interstadial-stadial pattern, although the leaf-wax records are somewhat less pronounced, probably due to a summer bias and the fact that the Younger Dryas was particularly a pronounced winter phenomenon. δ^2^H_*n*-C27_ is more enriched and variable, which likely reflects some transpirative and evaporative enrichment due to higher contributions from deciduous trees and aquatic sources, respectively.

δ^18^O_fucose_ is primarily of aquatic origin and reflects changes in δ^18^O of the lake water during summer. The typical stadial-interstadial pattern known from other δ^18^O records is not very obvious given the highly variable signal, but one cannot necessarily expect that pattern due to the summer bias of the proxy. Bichlersee is a small and closed basin, and it is sensitive to evaporative enrichment. Higher δ^18^O_fucose_ values during the Bølling–Allerød and Early Holocene than during the Younger Dryas may therefore indicate warmer temperatures and enhanced evaporation, but disentangling past changes in evaporative enrichment from changes in the isotopic composition of precipitation requires coupling δ^2^H and δ^18^O.

Our* d*-excess record basically shows the same pattern as δ^18^O_fucose_, which illustrates the high sensitivity of δ^18^O to evapo(transpi)rative enrichment compared to δ^2^H and highlights the enormous added value of δ^18^O_sugar_ analyses for paleohydrological reconstructions. The *d*-excess documents enhanced evaporative enrichment during the warm Bølling–Allerød and Early Holocene, and somewhat less enrichment during the Younger Dryas, which can be expected from lower temperatures. Furthermore, a long-term trend towards increasing enrichment from the Late Glacial to the Early Holocene follows and maybe explained with summer insolation.

In summary, our study highlights that isotope records have great potential for paleoclimate reconstructions. Site-specific hydrological conditions and seasonality need to be considered, and coupling δ^2^H and δ^18^O allows calculating *d*-excess and thus disentangling some of the many isotope effects.

## Methods

### Sampling and chronology

Two parallel cores were recovered from Bichlersee by using a piston coring system from UWITEC yielding a composite core length of 9.4 m in 2014. The cores were split in the lab, and one half was then subsampled at 1 cm intervals for further geochemical analyses.

Radiocarbon ages, mostly on macrofossils, were obtained in collaboration with the LARA AMS Laboratory at the University of Bern on a MIni CArbon DAting System (MICADAS) capable of direct analysis of CO_2_ due to the coupling to an Elementar Analyzer and a gas handling interface^83^. Prior to ^14^C analyses, all samples were treated with 1 M HCl for 8 h at 60 °C to remove carbonates. The samples were subsequently washed to pH neutrality with ultrapure water and weighed into tin boats. We performed Bayesian age-depth modelling using the ‘rBacon’ package (v. 2.5.8)^84^ available for R, and ^14^C ages were calibrated to cal. BP by applying the IntCal20 calibration curve^85^. Bayesian age-depth modelling was performed using the radiocarbon ages.

### Organic geochemistry and palynological analyses

All samples were dried at 40 °C and homogenized. To quantify total organic carbon (TOC), aliquots were treated with 1 M HCl and measured with a Total Organic Carbon Analyzer (Shimadzu TOC-V_CPN_) at Technische Universität Dresden.

Three samples from 495 cm, 497 cm, and 499 cm sediment depth were prepared for pollen analyses following the procedure described by Schneider, et al.^86^. This includes acetolysis and treatment with hydrofluoric acid. For pollen counting, the samples were mounted in silicon oil and analyzed using a Zeiss Light microscope at 400-times magnification.

### ***n***-Alkanes and compound-specific δ^2^H analyses

Total lipids were extracted at Friedrich-Schiller-Universität Jena with dichloromethane:methanol (DCM:MeOH, 9:1) from 80 samples (~ 0.1–12.6 g) using ultrasonic extraction over three cycles. The *n*-alkanes were cleaned over aminopropyl (Supelco, 45 µm) and silver-nitrate (AgNO_3_; Supelco, 60–200 mesh) columns and then quantified using a gas chromatograph (Agilent 7890B) equipped with an Agilent HP5MS column (30 m × 320 μm × 0.25 μm film thickness) and coupled with a flame ionization detector (GC-FID). External *n*-alkane standards (*n*-alkane mix *n*-C_21_ to *n*-C_40_; Supelco) were measured with each sequence for identification and quantification.

Compound-specific δ^2^H was analyzed for the most abundant homologues (*n*-C_27_, *n*-C_29_ and *n*-C_31_) using an Isoprime Vision isotope ratio mass spectrometer (IRMS; Elementar, Langenselbold) coupled to an Agilent 7890B gas chromatograph via a GC5 pyrolysis/combustion interface. The GC5 operated in pyrolysis mode with a Cr (ChromeHD) reactor at 1050 °C. All samples were injected in splitless mode and measured in triplicates. Analytical precision is on average 1.1‰ (standard deviation) and always < 3.2‰. Between the samples, a standard *n*-alkane mix with known isotopic values was measured, and all isotopic values are given in delta notation (δ^2^H_*n*-alkane_, ‰) versus ‘*Vienna Standard Mean Ocean Water*’ (V-SMOW). The ${\text{H}}_{3}^{+}$-correction factor was checked regularly throughout the sequences and yielded stable values of 3.2 ± 0.07‰ (*n* = 5).

### Hemicellulose sugars and compound-specific δ^18^O analyses

Hemicellulose sugars were extracted from 29 samples (up to ~ 600 mg, depending on TOC content) and processed according to Zech and Glaser^87^ at the Friedrich-Schiller-Universität Jena. The samples were extracted with 10 ml of 4 M trifluoroacetic acid at 105 °C for 4 h, cleaned using XAD-7 and Dowex 50WX8 columns and subsequently derivatized with methylboronic acid (1 mg in 100 ml pyridine) at 60 °C for 1 h. Myo-Inositol was used as internal standard.

Compound-specific δ^18^O of arabinose, fucose, and xylose was measured at the Technische Universität Dresden using a Trace GC 2000 coupled to a Delta V Advantage IRMS via an ^18^O-pyrolysis reactor (GC IsoLink) and a ConFlow IV interface (all Thermo Fisher Scientific, Germany). The samples were measured in triplicates, and standard blocks of derivatized sugars (arabinose, fucose, xylose) at various concentrations and known δ^18^O values were measured in between. Analytical precision is on average 0.5‰ (standard error) and always < 1.4‰. All measurements were corrected for hydrolytically introduced oxygen atoms that can form carbonyl groups within the sugar molecules^87^. The oxygen isotopic composition is given in the delta notation (δ^18^O_sugar_, ‰) versus V-SMOW.

### Supplementary Information

Supplementary Information.

## Data Availability

The datasets generated during the current study are available in the PANGAEA repository: https://doi.org/10.1594/PANGAEA.962035.
