# Supplementary material for: Summer paleohydrology during the Late Glacial and Early Holocene based on δ2H and δ18O from Bichlersee, Bavaria
Source: Sci Rep. 2023 Oct 28;13:18487. doi: 10.1038/s41598-023-45754-4 (PMC10613243; doi:10.1038/s41598-023-45754-4)
Supplement: Supplementary file 1 — Supplementary Information. [file 41598_2023_45754_MOESM1_ESM.pdf]

Supplementary Information to:

Summer paleohydrology during the Late Glacial and Early Holocene based on δ²H and δ¹⁸O from Bichlersee, Bavaria

Maximilian Prochnow[1], Paul Strobel[1], Marcel Bliedtner[1], Julian Struck[1], Lucas Bittner[2], Sönke Szidat[3], Gary Salazar[3], Heike Schneider[1], Sudip Acharya[1], Michael Zech[2], Roland Zech[1]

1 *Chair of Physical Geography, Institute of Geography, Friedrich Schiller University Jena, Jena, Germany*

2 *Heisenberg Chair of Physical Geography with Focus on Paleoenvironmental Research, Institute of Geography, Technical University of Dresden, Dresden, Germany*

3 *Department of Chemistry, Biochemistry and Pharmaceutical Sciences and Oeschger Centre for Climate Change Research, University of Bern, Bern, Switzerland*

Corresponding Author: Maximilian Prochnow (🖂: maximilian.prochnow@uni-jena.de)

S1. Model-based estimation of lake water deuterium excess

The combination of compound-specific δ^2H from *n*-alkanes and $\delta^{18}O$ from sugars in a "coupled isotope approach" allows the reconstruction of lake water deuterium excess (*d*-excess), which is a valuable proxy for lake water evaporative enrichment[1]. With $\delta^{18}O_{fucose}$ and $\delta^2H_{n\text{-}C31}$, our Bichlersee record provides only one robust aquatic and terrestrial signal, respectively. However, the concept of the "coupled isotope approach" requires both isotopes to reflect an aquatic signal to reconstruct *d*-excess.

Here, we describe a potential "work-around" based on the coupled isotope approach and show how *d*-excess of lake water can be estimated when a second aquatic component is absent. A graphical overview of this mathematical approach is shown in Supplementary Figure 1.

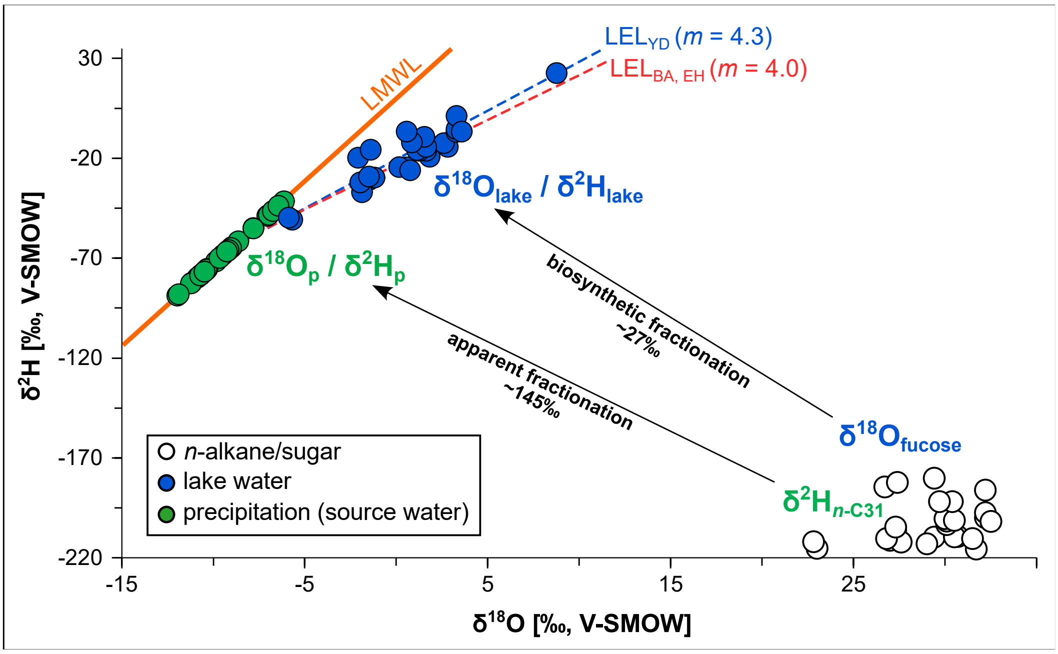

Supplementary Figure 1: The graphic shows the calculated $\delta^{18}O$ and δ^2H for lake water and precipitation, respectively, within the $\delta^{18}O$-δ^2H-diagram. Arrows indicate how δ^2H_p and $\delta^{18}O_{lake}$ was calculated. Two different LELs and their slope *m* are shown for the Bølling-Allerød (BA)/Early Holocene (EH) and the Younger Dryas (YD).

Calculating lake water $\delta^{18}O$ and precipitation $\delta^{2}H$

First, the aquatic $\delta^{18}O_{fucose}$ can be transformed to lake water $\delta^{18}O$ ($\delta^{18}O_{lake}$) by applying the known biosynthetic fractionation (ε_{bio}) of 27‰ for hemicellulose sugars[2,3]:

$$\delta^{18}O_{lake} = \left[\frac{\left(\frac{\delta^{18}O_{fucose}}{1000-1}\right)}{\left(\frac{\varepsilon_{bio}}{1000-1}\right)} - 1\right] \times 1000 \quad (1)$$

Second, we derive $\delta^{2}H$ of precipitation ($\delta^{2}H_{p}$) from terrestrial grass-derived $\delta^{2}H_{n\text{-}C31}$ by applying a constant apparent fractionation factor (ε_{app}) between $\delta^{2}H_{n\text{-}C31}$ and $\delta^{2}H_{p}$:

$$\delta^{2}H_{p} = \left[\frac{\left(\frac{\delta^{2}H_{n\text{-}C31}}{1000-1}\right)}{\left(\frac{\varepsilon_{app}}{1000-1}\right)} - 1\right] \times 1000 \quad (2)$$

We use an apparent fractionation factor of −145±12‰ for grass sites following a European transect study published by Hepp, et al. [4]. Combined with the equation of the local meteoric waterline (LMWL) for Garmisch-Partenkirchen (equation 3)[5], this further allows to estimate the corresponding $\delta^{18}O$ values of precipitation ($\delta^{18}O_{p}$; equation 4):

$$\delta^{2}H_{p} = 8.14 \times \delta^{18}O_{p} + 9.05 \quad (3)$$

$$\delta^{18}O_{p} = \frac{(\delta^{2}H_{p} - 9.05)}{8.14} \quad (4)$$

Estimating lake water $\delta^{2}H$ and *d*-excess

To calculate $\delta^{2}H_{lake}$ and *d*-excess, a local evaporation line (LEL) with function

$$\delta^{2}H_{lake} = m * \delta^{18}O_{lake} + n \quad (5)$$

must be inferred, where m is the LEL's slope and n its parameter defining the intersection with the $\delta^{2}H$-axis in the $\delta^{18}O$-$\delta^{2}H$-diagram. Equilibrium factors for ^{18}O and ^{2}H can be calculated after Horita and Wesolowski [6] using documented paleotemperatures for the Younger Dryas, Bølling-Allerød and Early Holocene (STab. 1). Therefore, we calculated a paleotemperature for all three climate periods relative to modern MAT by assuming a temperature amplitude in the order of ~4 °C during the Bølling-Allerød-Younger Dryas-Early Holocene transition[7,8]. Due to the higher altitude of Bichlersee (960 m a.s.l.) compared to Kiefersfelden (520 m a.s.l.), we corrected the MAT of Kiefersfelden (8.8 °C) for the hypsometric temperature gradient of –0.6 m * 100 m^{-1}, yielding a MAT for Bichlersee of ~6.2 °C.

Supplementary Table 1: Paleotemperatures, equilibrium factors for ^{18}O and ^{2}H and slopes for the Local evaporation lines (LELs) applied in the coupled-isotope approach.

Climate period	Calculated Paleotemperature	Equilibrium factor for ^{18}O	Equilibrium factor for ^{2}H	Slope *m* of LEL
Early Holocene	~10.2 °C	10.57‰	88.8‰	4.0
Younger Dryas	~2.2 °C	11.36‰	98.2‰	4.3
Bølling-Allerød	~10.2 °C	10.57‰	88.8‰	4.0

The kinetic fractionation factor for ^{2}H and ^{18}O is set to 12.4‰ and 14.3‰, respectively[9]. This yields slightly different slopes for the LEL during the Bølling-Allerød, the Younger Dryas, and the Early Holocene (STab. 1). Because the LEL intersects the LMWL, the parameter n can be derived for every data point using $\delta^{18}O_{p}$ and $\delta^{2}H_{p}$:

$n = \delta^2H_p - (m \times \delta^{18}O_p)$ (6)

After $\delta^{18}O_{lake}$, the slope *m* and parameter *n* as the intersect with δ^2H for the LEL is obtained, we are now able to derive δ^2H_{lake}:

$\delta^2H_{lake} = m \times \delta^{18}O_{lake} + n$ (7)

With $\delta^{18}O_{lake}$ and δ^2H_{lake}, equation 8 can be used to calculate *d*-excess of lake water:

$d\text{-excess} = \delta^2H_{lake} - (8.14 \times \delta^{18}O_{lake})$ (8)

Potential limitations

In our study, we used the apparent fractionation of –145±12‰ for grass sites suggested by a European transect study carried out by Hepp, et al. [4]. This assumption seems reasonable because very similar values of –142±16‰ were also reported for grass-sites in Mongolia[10,11]. However, this generalization is a potential uncertainty in our approach because several other studies, investigating the apparent fractionation of $\delta^2H_{n\text{-C31}}$ and δ^2H of precipitation, reveal a comparatively large scatter up to several tens of permille[12]. This scatter was previously related to analytical uncertainties or age-offsets between biomarker compounds and environmental signals, whereas different vegetation patterns and plant-type specific biosynthetic effects can also have an influence[12]. Therefore, different apparent fractionation factors would in fact change the absolute values of *d*-excess in our approach, but its general trend should remain the same.

For this approach, a LMWL derived from modern precipitation in Garmisch-Partenkirchen was used for the calculation of *d*-excess. However, the relationship of $\delta^{18}O_p$ and δ^2H_p in paleoprecipitation could have changed, as it is known from *d*-excess reconstructions from Greenland, where *d*-excess varies up to ~5‰ during the Lateglacial[13]. Moreover, *d*-excess in modern precipitation at Bichlersee shows a seasonal variability of only ~4‰ (STab. 2). However, the amplitude of these variations is much smaller compared to the reconstructed variability of our lake water *d*-excess (~40‰), and we therefore suggest that this uncertainty has only a minor influence on our *d*-excess reconstruction.

Supplementary Table 2: Seasonal variability of stable water isotopes and *d*-excess at Bichlersee[14-16].

	Jan	Feb	Mar	Apr	May	Jun	Jul	Aug	Sept	Oct	Nov	Dec
**δ^2H_p [‰, V-SMOW]**	-108	-107	-95	-76	-54	-45	-33	-37	-45	-62	-88	-101
**$\delta^{18}O_p$ [‰, V-SMOW]**	-15	-15	-13	-11	-8	-7	-5	-6	-7	-9	-12	-14
***d*-excess [‰] (based on LMWL)**	12.5	12.7	12.4	11.1	11.1	10.4	9.3	11.8	11.2	12.1	13.0	13.0

Moreover, the slopes of the LELs derived for the Younger Dryas and Bølling-Allerød are dependent on temperature. Therefore, reconstructed paleotemperatures based on chironomids[7,8] were used to calculate the slope *m* of the LELs. We point out that temperature patterns in the Alps can be regionally different, and unfortunately, there is no local temperature reconstruction for the Bavarian Mangfall Mountains available. This does indeed introduce some additional uncertainty in the *d*-excess calculations. However, according to the calculations of Horita and Wesolowski [6] and Araguás-Araguás, et al. [9], the sensitivity of the LEL's slope *m* to temperature is only ~ –0.03 per 1 °C (SFig. 2).

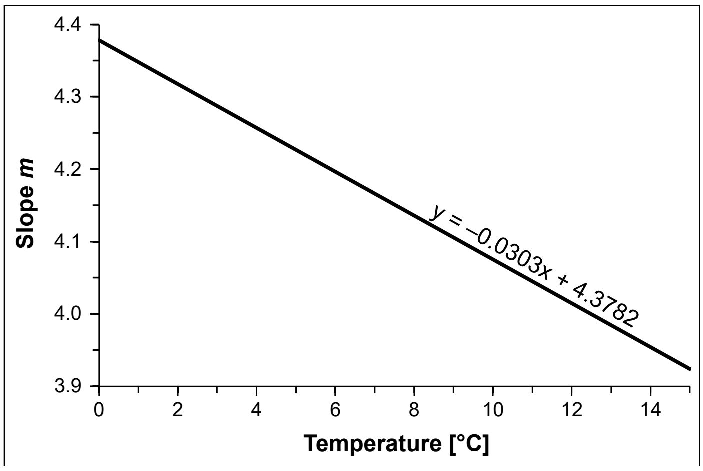

Supplementary Figure 2: Temperature versus slope *m* of the local evaporation line according to Horita and Wesolowski [6] and Araguás-Araguás, et al. [9].

The chironomid-based temperature reconstructions by Heiri, et al. [7] and Ilyashuk, et al. [8] applied in this study generally agree well with each other and moreover, uncertainties in the absolute temperature values used for slope calculation should not have a strong impact on the resulting *d*-excess. We also acknowledge that the resulting *d*-excess is only a broad approximation for lake water evaporative enrichment and should not be quantitatively interpreted at Bichlersee yet.

S2. Pollen data

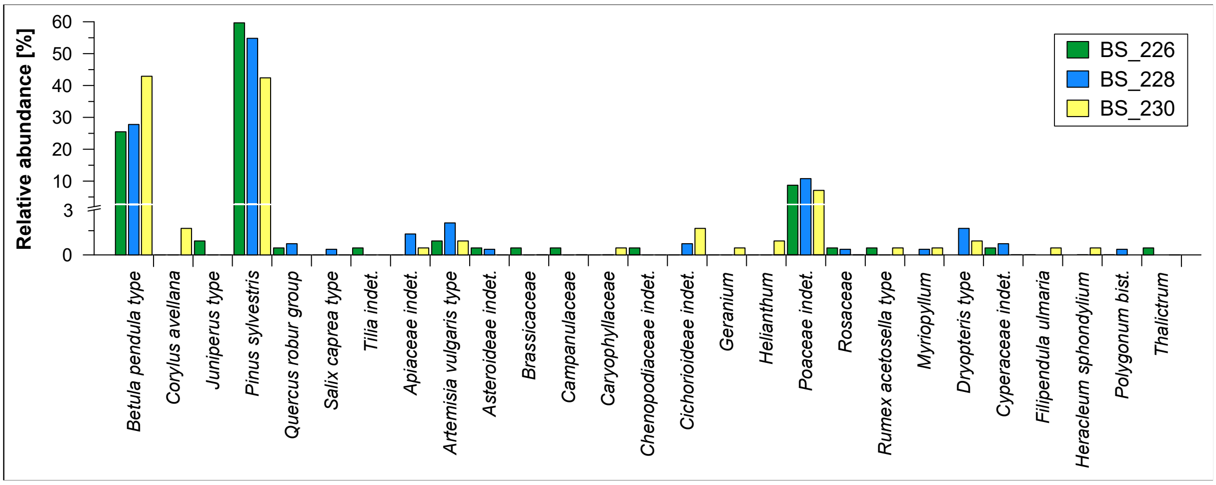

Supplementary Figure 3: Relative abundances of pollen investigated in the samples BS_226 (495 cm), BS_228 (497 cm) and BS_230 (499 cm).

S3. Relative abundances of *n*-alkanes

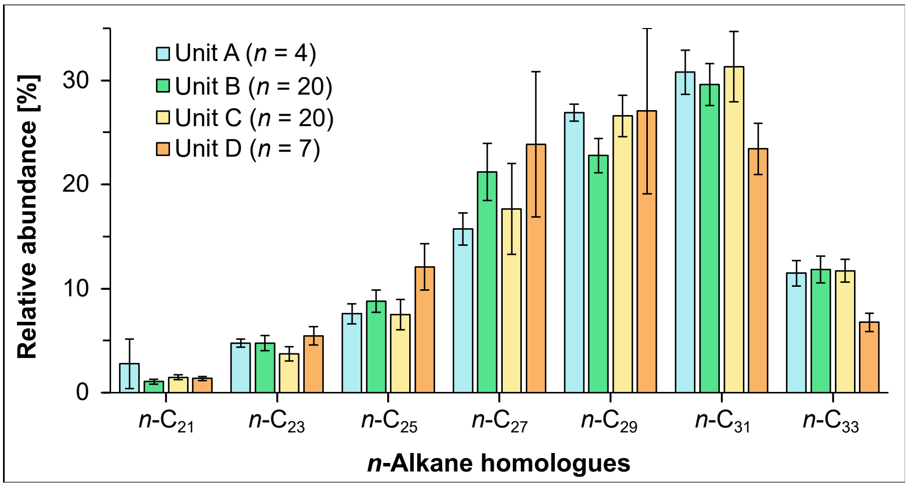

Supplementary Figure 4: Relative abundances of *n*-alkanes (*n*-C_{21} to *n*-C_{33}) for Unit A to D of the Bichlersee record.

Supplementary References

1 Hepp, J. *et al.* Reconstructing lake evaporation history and the isotopic composition of precipitation by a coupled $\delta^{18}O$–$\delta^{2}H$ biomarker approach. *Journal of Hydrology* **529**, 622–631; 10.1016/j.jhydrol.2014.10.012 (2015).

2 Gessler, A. *et al.* Tracing carbon and oxygen isotope signals from newly assimilated sugars in the leaves to the tree-ring archive. *Plant, Cell & Environment* **32**, 780-795; 10.1111/j.1365-3040.2009.01957.x (2009).

3 Cernusak, L. A., Wong, S. C. & Farquhar, G. D. Oxygen isotope composition of phloem sap in relation to leaf water in Ricinus communis. *Functional Plant Biology* **30**, 1059-1070; 10.1071/FP03137 (2003).

4 Hepp, J. *et al.* Evaluation of bacterial glycerol dialkyl glycerol tetraether and ^{2}H–^{18}O biomarker proxies along a central European topsoil transect. *Biogeosciences* **17**, 741–756; 10.5194/bg-17-741-2020 (2020).

5 IAEA/WMO. *Global Network of Isotopes in Precipitation. The GNIP Database.*, <https://nucleus.iaea.org/wiser> (2019).

6 Horita, J. & Wesolowski, D. J. Liquid-vapor fractionation of oxygen and hydrogen isotopes of water from the freezing to the critical temperature. *Geochimica et Cosmochimica Acta* **58**, 3425-3437; 10.1016/0016-7037(94)90096-5 (1994).

7 Heiri, O., Ilyashuk, B., Millet, L., Samartin, S. & Lotter, A. F. Stacking of discontinuous regional palaeoclimate records: Chironomid-based summer temperatures from the Alpine region. *The Holocene* **25**, 137-149; 10.1177/0959683614556382 (2015).

8 Ilyashuk, B. *et al.* Lateglacial environmental and climatic changes at the Maloja Pass, Central Swiss Alps, as recorded by chironomids and pollen. *Quaternary Science Reviews* **28**, 1340–1353; 10.1016/j.quascirev.2009.01.007 (2009).

9 Araguás-Araguás, L., Froehlich, K. & Rozanski, K. Deuterium and oxygen-18 isotope composition of precipitation and atmospheric moisture. *Hydrological Processes* **14**, 1341-1355; 10.1002/1099-1085(20000615)14:8%3C1341::AID-HYP983%3E3.0.CO;2-Z (2000).

10 Strobel, P., Struck, J., Zech, R. & Bliedtner, M. The spatial distribution of sedimentary compounds and their environmental implications in surface sediments of Lake Khar Nuur (Mongolian Altai). *Earth Surface Processes and Landforms* **46**, 611–625; 10.1002/esp.5049 (2021).

11 Struck, J. *et al.* Leaf Waxes and Hemicelluloses in Topsoils Reflect the $\delta^{2}H$ and $\delta^{18}O$ Isotopic Composition of Precipitation in Mongolia. *Frontiers in Earth Science* **8**; 10.3389/feart.2020.00343 (2020).

12 Struck, J. *Calibration and first application of biomarker and compound-specific isotope analyses in Mongolia* PhD thesis, Friedrich-Schiller-Universität, (2022).

13 Masson-Delmotte, V. *et al.* GRIP Deuterium Excess Reveals Rapid and Orbital-Scale Changes in Greenland Moisture Origin. *Science* **309**, 118-121; 10.1126/science.1108575 (2005).

14 Bowen, G. J. *The Online Isotopes in Precipitation Calculator*, <http://www.waterisotopes.org> (2023).

15 Bowen, G. J. & Revenaugh, J. Interpolating the isotopic composition of modern meteoric precipitation. *Water Resources Research* **39**, 23; 10.1029/2003wr002086 (2003).

16 Bowen, G. J., Wassenaar, L. I. & Hobson, K. A. Global application of stable hydrogen and oxygen isotopes to wildlife forensics. *Oecologia* **143**, 337-348; 10.1007/s00442-004-1813-y (2005).
